# Combined Therapy of Catheter Ablation and Left Atrial Appendage Closure for Patients with Atrial Fibrillation: A Case-Control Study

**DOI:** 10.1155/2020/8615410

**Published:** 2020-06-25

**Authors:** Bin-Feng Mo, Jian Sun, Peng-Pai Zhang, Wei Li, Mu Chen, Jia-Li Yuan, Yi Yu, Qun-Shan Wang, Yi-Gang Li

**Affiliations:** Department of Cardiology, Xinhua Hospital Affiliated to Shanghai Jiao Tong University School of Medicine, #1665 Kong Jiang Road, Shanghai 200092, China

## Abstract

**Aim:**

The feasibility and safety of performing the combined procedure of catheter ablation (CA) and left atrial appendage closure (LAAC) for atrial fibrillation (AF) have been reported by observational studies without controls. The aim of this study was to compare the procedural and long-term outcomes of combined procedures with isolated CA or LAAC.

**Methods and Results:**

This study included patients who underwent combined CA and LAAC (combined group), CA alone (CA-only group), or LAAC alone (LAAC-only group). Propensity score matching was used to select controls from the CA-only and LAAC-only groups. Each group contained 76 subjects. The procedures were successfully performed in all the patients. Procedure-related complications of the combined group included one pericardial effusion and two groin haematomas, which did not differ significantly with those of the CA-only group (3.9% vs. 2.6%, *P*=0.650) or the LAAC-only group (3.9% vs. 2.6%, *P*=0.650), respectively. The AF-free rate of the combined group was comparable with that of the CA-only group after a mean of 2 years follow-up (67.1% vs. 69.7%, *P*=0.727). Compared with the LAAC-only group, the combined group achieved similar complete occlusion rate at implant (94.7% vs. 93.4%) and at 45 days (82.9% vs. 85.5%). At the end of follow-up, ischemic stroke and bleeding events of the combined group were low (3.9%) and were comparable with those of the CA-only group (5.3%) and the LAAC-only group (2.6%).

**Conclusions:**

The combination of AF-CA and LAAC is safe and efficacious compared with single procedures alone.

## 1. Introduction

Catheter ablation (CA) is more effective than antiarrhythmic drugs to achieve rhythm control for symptomatic atrial fibrillation (AF), with additional benefits of reducing arrhythmic burden and improving quality of life [1–3]. However, the role of AF ablation in long-term stroke prevention has not been well established [4]. In patients with increased risk of stroke and bleeding, left atrial appendage closure (LAAC) has been proven a safe and effective alternative to long-term anticoagulation [5]. Recently, combined procedures of CA for AF and LAAC for stroke prevention have raised increasing attention in selected patients with high risk of stroke and bleeding. Observational studies without controls have reported the feasibility and safety of performing combined procedures [6–12]. However, clinical outcomes on procedural safety, AF recurrence, and adverse events of the combined procedure compared with those of the single procedures have not been reported. Thus, the aim of this study was to compare the procedural and long-term outcomes of the combined procedure with isolated CA and LAAC.

## 2. Methods

### 2.1. Study Population

**Figure 1:**
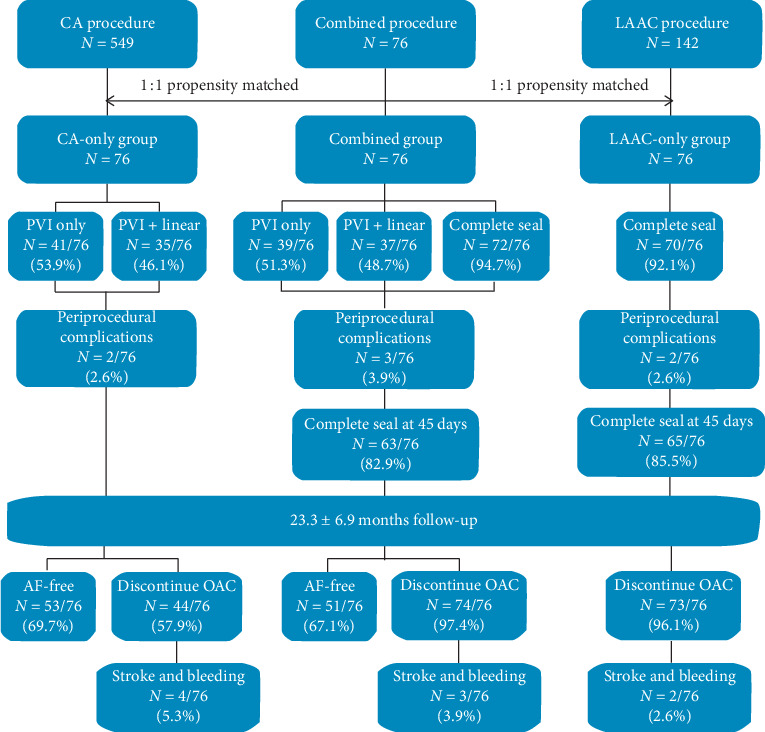
Study flowchart. Flowchart showing the study design and the procedural and long-term outcomes in the three groups. AF, atrial fibrillation; CA, catheter ablation; LAAC, left atrial appendage closure; OAC, oral anticoagulation; PVI, pulmonary vein isolation.

This single-center retrospective study enrolled consecutive patients with nonvalvular AF between April 2017 and September 2018. All participants were included based on the following criteria: (1) age >18 years undergoing the first CA and/or LAAC procedure, (2) indications for CA were symptomatic nonvalvular AF refractory to antiarrhythmic drugs, and (3) all patients who underwent LAAC met at least one of the following indications: (i) CHA_2_DS_2_-VASc scores ≥2 and HAS-BLED scores ≥3; (ii) intolerance to chronic oral anticoagulation (OAC); and (iii) stroke/TIA or thromboembolism (TE) even under OAC treatment [4, 13, 14]. A total of 76 patients underwent combined procedures who constituted the combined group. During the same period of time, isolated CA or isolated LAAC procedures were performed in 549 and 142 patients, respectively. Propensity score matching was used to select controls to minimize potential confounding bias. Patients of the combined group were matched 1 : 1 to those who underwent isolated CA (CA-only group) and to those who underwent isolated LAAC (LAAC-only group), respectively. A total of 228 patients in three groups were included in the final analysis (Figure 1). This retrospective study was approved by the Ethics Committee of Xinhua Hospital affiliated to Shanghai Jiao Tong University School of Medicine and complied with the Declaration of Helsinki. Written informed consent was obtained from each patient.

### 2.2. Preprocedural Assessment

All the procedures were performed in a high-volume AF center (>1000 cases of AF intervention per year) and undertaken by experienced operators who had passed the learning curves of either CA or LAAC. Left atrial appendage (LAA) thrombus exclusion and size measurement were conducted by transesophageal echocardiography (TEE) before procedures. A cardiac computed tomography (CT) scan and 3-dimensional reconstruction of the left atrium were performed before procedure in 82% (187/228) of patients to assist CA and/or LAAC.

### 2.3. Catheter Ablation Procedure

Under conscious sedation, a decapolar catheter was positioned in the coronary sinus, and two transseptal accesses were obtained through the right femoral vein. Mapping and ablation were performed under the guidance of CARTO (Biosense Webster, Diamond Bar, CA, USA) or EnSite (St. Jude Medical, St Paul, MN, USA) 3-dimensional electroanatomic mapping systems in addition to standard fluoroscopy. For patients with paroxysmal AF, standard pulmonary vein isolation (PVI) was performed, and for those with persistent AF, additional linear and/or complex fragmented atrial electrogram (CFAE) ablations were performed according to the physician's discretion. Sinus rhythm was restored by either ablation or electric cardioversion.

### 2.4. Left Atrial Appendage Closure Procedure

LAAC procedure was performed under local anesthesia, and TEE was introduced under deep sedation after device deployment to reconfirm the position of the device before release. The procedure was performed as described previously [15]. In brief, the ostial width and depth of the LAA were measured by LAA angiography or cardiac CT images. A mean left atrial pressure above 10 mmHg was obtained before measurement. Only WATCHMAN^TM^ (Boston Scientific, MA, USA) devices were used. The device with appropriate size was chosen when the depth was allowed, generally 3–6 mm oversizing based on the maximum diameter [16]. LAAC implantation was performed after AF ablation was completed in the combined group. We preferred a relatively larger device in the combined procedure since pulmonary vein ridge edema may occur after ablation and in case of the novel leak after the procedure. The device was then advanced into the delivery sheath and deployed by sheath retraction guided by fluoroscopy. Preliminary assessment was performed by angiography and TUG test under fluoroscopy to check the device position and stability. TEE was then performed to reconfirm the position with minimal (<5 mm) to no residual peridevice leaks and a proper compression ratio under deep sedation. The device was released if it was verified by the assessment of “PASS” criteria.

### 2.5. Postprocedural Anticoagulation

In each group, patients received OAC therapy for at least 3 months following the procedure, unless there were contraindications. In the CA-only group, life-long OAC was recommended for those with a CHA_2_DS_2_-VASc score ≥2, and OAC was discontinued for those with a CHA_2_DS_2_-VASc score <2 if no AF/AT recurrence was observed 6 months following the procedure [4]. For patients in the combined group and the LAAC-only group, dual antiplatelet therapy was recommended for another 3 months, and then life-long aspirin was prescribed if follow-up TEE showed either complete closure of the LAA or limited residual peridevice flow (jet <5 mm in width) [14].

### 2.6. Follow-Up

After discharge, office or transtelephonic visits were scheduled at the 3rd month, 6th month, and 12th month following the procedure and once half a year thereafter. ECG or 24 h Holter monitoring was performed at each office visit for patients of the combined group and the CA-only group. Antiarrhythmic drug therapy was discontinued after 3 months if no clinical or documented AF recurrences were identified. In the combined group and the LAAC-only group, TEE was performed to assess the device occlusion safety and efficiency at 45 days and 6 months of follow-up time points according to the percutaneous LAA occlusion Munich Consensus Document [14]. New peridevice leaks were defined as newly detected leaks during the TEE follow-up that were not observed immediately after the device was implanted. Persistent leaks indicated unresolved peridevice leaks from implantation till reassessment.

### 2.7. Statistical Analysis

Propensity score matching was used to select controls to minimize potential confounding bias. The propensity scores were constructed using the following variables: age, sex, AF type, hypertension, heart failure, diabetes mellitus, coronary artery disease, and previous stroke/TIA/TE. We conducted propensity score matching using nearest neighbor matching, and no matching samples were removed. Patients of the CA-only group and the LAAC-only group were matched to the combined group at a 1 : 1 ratio with a caliper width of 0.10 and 0.18 standard deviations of the propensity score, respectively.

Continuous variables are described as mean ± standard deviation (median (interquartile range) for nonnormal data) and are compared using Student's *t*-test (Mann–Whitney *U* test if normality not satisfied). Categorical variables are presented as percentages and are analyzed using chi-square test or Fisher exact test where appropriate. Variables of the combined group were compared with those of the CA-only and the LAAC-only group, respectively. All analyses were performed using SPSS version 22.0 (IBM Software Inc., Armonk, NY). Two-sided *P* value of <0.05 was considered statistically significant.

## 3. Results

### 3.1. Baseline Characteristics

Baseline characteristics before propensity score matching are shown in Table 1. Compared with the combined group, patients in the CA-only group were younger and more likely to have paroxysmal AF and a smaller left atrium but less likely to have heart failure and previous stroke/TIA/TE. The CHA2DS2-VASc and HAS-BLED scores of the CA-only group were both lower than those of the combined group. Compared with the combined group, patients in the LAAC-only group were older and more likely to have persistent AF and a larger left atrium.

After propensity score matching, three groups with each of 76 subjects were matched (Table 2). There were no significant differences in age, AF type, left atrium size, CHA2DS2-VASc score, and comorbidities with heart failure and previous stroke/TIA/TE between the CA-only group and combined group after matching. The HAS-BLED score of the CA-only group was still lower than that of the combined group (2.6 ± 0.9 vs. 3.3 ± 1.1, *P* < 0.001). The baseline characteristics were comparable between the combined group and the LAAC-only group after matching.

### 3.2. Catheter Ablation and Arrhythmia Follow-Up

The periprocedural outcomes of catheter ablation are given in Table 3. A total of 39 patients (51.3%) of the combined group and 41 patients (53.9%) of the CA-only group underwent PVI only (*P*=0.745). Additional linear/CFAE ablations were performed in the rest of the patients. The procedure time (129 ± 24.5 min vs. 133.1 ± 26.4 min) and fluoroscopy time (5.4 ± 2.4 min vs. 5.9 ± 2.6 min) were comparable between the combined group (not including the time for LAAC) and the CA-only group. There were three patients (3.9%) with procedure-related complications in the combined group. One was pericardial effusion which required percutaneous drainage, and the other two were minor groin haematomas. In the CA-only group, one patient had pericardial effusion and one patient had a groin haematoma. No significant difference was observed in the procedure-related complications between the two groups (*P*=0.650).

After a mean of 24 months follow-up, 51 patients (67.1%) in the combined group and 53 patients (69.7%) in the CA-only group were AF-free of antiarrhythmic drugs (*P*=0.727) (Table 3). Twelve patients (15.8%) in the combined group and nine patients (11.8%) in the CA-only group had AF recurrence and underwent a repeat ablation.

### 3.3. LAAC and TEE Follow-Up

The morphology of the LAA was equally distributed between the combined group and the LAAC-only group (Table 4). The device size used in the combined group was larger according to the ostium width of the LAA, which resulted in obvious larger (but not significantly) device compression (19.6 ± 4.5% vs. 18.4 ± 4.3%, *P*=0.085) between the groups. All patients of the two groups achieved a satisfactory seal (residual leak ≤5 mm). Complete occlusion was achieved in 94.7% of the combined group and 93.4% of the LAAC-only group (*P*=0.513). The procedure time and fluoroscopy time calculated from the WATCHMAN access sheath exchange to the end of the procedure were not significantly different between the two groups (*P*=0.149 and 0.273, respectively). Three patients (3.9%) in the LAAC-only group had procedure-related complications. One suffered transient coronary air embolism with chest pain and ST segment elevation in inferior wall leads, which was resolved by forced coughing. The other one was minor groin haematoma. There was no significant difference in complications between the combined group and the LAAC-only group (3.9% vs. 2.6%, *P*=0.650).

Data on TEE imaging at least 45 days after the procedure were available in 74 patients (97.3%) of the combined group and 73 patients (96.1%) of the LAAC-only group. Five patients were evaluated by CT imaging. Proper seal (residual leak ≤5 mm) was found in all the patients, and device-related thrombosis (DRT) was detected on TEE in one patient (1.3%) of the LAAC-only group resolving without clinical sequelae on continued oral anticoagulation. Patients with complete occlusion (82.9% vs. 85.5%, *P*=0.656), new peridevice leaks (13.2% vs. 9.2%, *P*=0.440), and persistent peridevice leaks (3.9% vs. 1.3%, *P*=0.311) were comparable between two groups at 45 days follow-up time point (Table 4).

### 3.4. Antithrombotic Therapy and Clinical Outcomes

**Figure 2:**
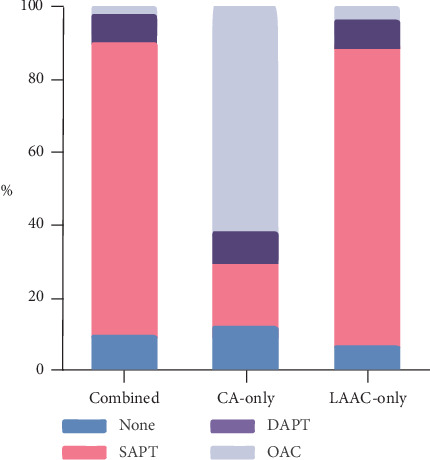
Antithrombotic treatment of each group at the end of follow-up. Most of the combined group and the LAAC-only group discontinued OAC, while 61.8% of the CA-group continued on OAC. DAPT, dual antiplatelet therapy; OAC, oral anticoagulation; SAPT, single antiplatelet therapy.

After the procedure, 100.0% of the CA-only group, 96.0% of the combined group, and 97.4% of the LAAC-only group were prescribed an OAC, while the rest of the patients were given antiplatelet therapy. During the latest follow-up, 2.6% of the combined group remained on OAC, while antiplatelet was prescribed in 88.2% (80.3% single and 7.9% dual), and the remainder 9.2% received no therapy (Figure 2). The distribution of antithrombotic therapy in the LAAC-only group was similar to the combined group (Figure 2). The rates of antithrombotic therapy in the CA-only group were as follows: 61.8% OAC, 26.3% antiplatelet therapy (17.1% single and 9.2% dual), and 11.8% on no therapy.

In the combined group, a total of two patients had bleeding events (one pulmonary and one epistaxis), and one had ischemic stroke during follow-up. The stroke happened in the seventh month after implantation, and the patient was recommenced to change from antiplatelet therapy back to OAC thereafter. There was no evidence for device thrombus in this patient. The event rate for the CA-only group and the LAAC-only group was 5.3% (3 bleeding and 1 ischemic stroke) and 2.6% (2 bleeding and 0 ischemic stroke), respectively (Tables 3 and 4). The event rates of the CA-only group and the LAAC-only group did not differ significantly from those of the combined group (*P*=0.699and *P*=0.650).

## 4. Discussion

The current study presents a comparison between combined procedures and the isolated CA or LAAC procedures. By comparing the single procedures, the combined therapy (i) does not introduce additional periprocedural complications; (ii) has comparable rate of AF recurrence and successful LAA occlusion, and (iii) provides satisfactory outcomes of stroke prevention and bleeding events during a mean of 2 years of follow-up. The results support and supplement the safety and efficacy of the combined procedure of AF ablation and LAAC, which were previously published in noncontrol studies [8–12].

Catheter ablation for AF has been proven to improve symptoms and quality of life, but no randomized clinical trial has shown a reduction in long-term ischemic stroke. [1] On the contrary, percutaneous LAAC, especially with WATCHMAN device, has been demonstrated in randomized trials to reduce stroke and therefore can be an alternative to warfarin therapy for stroke prevention [17, 18]. The combination of LAAC with CA is an effective way to ameliorate the symptoms of AF, while at the same time, reducing the risk of stroke, which was first reported by Swaans et al. in a small observational study of 30 patients [6]. Since then, a number of studies, from single-center experiences to multicenter registry results, support and strengthen that the combined therapy can be feasible, safe, and successful [7–12]. However, all these observational studies lack control groups. In this study, we selected a CA-alone group and a LAAC-alone group by the propensity matching score as control groups for combined therapy. Our results further confirmed favorable outcomes of the combined CA and LAAC therapy in the aspect of safety, AF recurrence, and adverse events.

### 4.1. Periprocedural Safety

Although AF ablation or LAAC alone has been shown to be safe and effective, concomitant intervention of these two procedures remains to be concerned about whether there are additional periprocedural risks. In this study, the procedure-related complications included one pericardial effusion and two groin haematomas but no death. Compared with CA alone (2.6%) and LAAC alone (2.6%), combined therapy (3.9%) did not significantly increase procedure-related complications. The procedural-related complication/serious adverse event rate of combined therapy reported by two multicenter studies was 6.2% and 2.1% [9, 12], which was consistent with the results of this study. A Chinese multicenter registry with the same population of our study documented a similar rate of 4.1% [11]. Our results support good periprocedural safety of the combined procedure.

### 4.2. Assessment of AF Recurrence

The impact of LAAC on the rhythm outcomes of AF ablation is still not clear. A small randomized trial suggested that the combination of LAAC with CA did no influence on the long-term AF recurrences [19]. In this case-control study, the AF-free rate of the combined group was comparable with that of the CA-only group after nearly 2 years of follow-up. No differences of recurrence were also observed in subgroups of paroxysmal and persistent AF either. Of note, the presence of the WATCHMAN device did not appear to interfere when doing a repeat ablation. Larger randomized trials are needed to assess whether the use of LAA occlusion devices will impact the outcomes of AF ablation.

### 4.3. Assessment of LAA Occlusion

The complete occlusion rate of the combined group decreased from 94.7% at implant to 82.9% at 45 days follow-up. Combined procedural data on CA and LAAC of the evolution and wasp studies published by Phillips et al. showed reduction in complete sealing from 97.1% to 61.0% at the first follow-up [8]. Wintgens et al. reported a similar reduction in a multicenter study [9]. Pulmonary vein ridge edema, which may lead to underestimation of the true LAA diameter, was the main concern in combined therapy. In our experience, a larger device size or measurement of the LAA diameter by preprocedure CT may reduce this risk. However, a similar reduction in complete occlusion was observed in the LAAC-only group. The PROTECT AF trial with LAAC procedure alone also demonstrated a peridevice leak rate of 40.9% at 45 days follow-up [20]. Explanations and clinical consequences of the new peridevice leaks require further investigation.

### 4.4. Antithrombotic Therapy and Clinical Follow-Up

LAAC with WATCHMAN device has been demonstrated superior to warfarin in randomized trials to reduce stroke and bleeding risk [17]. In our study, more than 95% of patients in the combined group and the LAAC-only group discontinued OAC, while more than 60% of the CA-only group continued on OAC. The stroke and bleeding event rates of the combined and LAAC-only groups were similar to those of the CA-only group, although patients of the prior groups had higher bleeding risk and discontinued OAC after 3 months of the procedure. The efficacy of combined therapy in reduction of stroke and bleeding compared with the calculated risk based on CHA2DS2-VASc and HAS-BLED scores was also reported by the two multicenter studies [9, 12].

### 4.5. Limitations

This was a single-center retrospective study with a moderate sample size. Because the cohort consists of three different nonrandomized groups, a selection bias may exist. We attempted to minimize the selection bias by using a propensity score to select patients to be included into the CA-only and LAAC-only groups. Future larger randomized trials are required to compare combined therapy with CA alone or LAAC alone. The follow-up method of ECG and 24 h Holter recordings for detecting AF recurrence is another limitation. Asymptomatic arrhythmias or nondocumented symptomatic episodes might have been missed.

## 5. Conclusions

The combination of AF-CA and LAAC is safe and efficacious compared with single procedures alone. Randomized control trials are needed to further verify the benefit of combined therapy in selected patients with AF.

## Figures and Tables

**Table 1:** Baseline characteristics of the study population before propensity score matching.

	Combined (*n* = 76)	CA-only (*n* = 549)	*P* ^*∗*^	LAAC-only (*n* = 142)	*P* ^†^
Female	37 (48.7)	285 (51.9)	0.598	67 (47.2)	0.833
Age (years)	69.9 ± 7.9	65.2 ± 7.8	<0.001	72.7 ± 8.4	0.019
BMI (kg/m^2^)	24.6 ± 3.2	24.9 ± 3.9	0.551	24.1 ± 3.0	0.274
Paroxysmal AF	37 (48.7)	344 (62.7)	0.019	30 (21.1)	<0.001
Persistent AF	39 (51.3)	205 (37.3)	0.019	112 (78.9)	<0.001
Coronary artery disease	16 (21.1)	74 (13.5)	0.246	37 (26.1)	0.204
Hypertension	56 (73.7)	358 (65.2)	0.143	118 (83.1)	0.099
Heart failure	23 (30.3)	68 (12.4)	<0.001	36 (25.4)	0.437
Diabetes mellitus	14 (18.4)	87 (15.8)	0.568	37 (26.1)	0.204
Previous stroke/TIA/TE	23 (30.3)	63 (11.5)	<0.001	35 (24.6)	0.371
Left atrial diameter (mm)	42.7 ± 5.7	38.4 ± 5.5	<0.001	44.7 ± 6.0	0.023
LVEF (%)	63.9 ± 6.3	64.8 ± 6.8	0.271	62.2 ± 7.4	0.104
CHA_2_DS_2_-VASc score	3.6 ± 1.3	2.5 ± 1.5	<0.001	3.9 ± 1.3	0.122
HAS-BLED score	3.3 ± 1.1	1.7 ± 0.9	<0.001	3.5 ± 1.1	0.105

*∗*Comparison between the combined group and the CA-only group. ^**†**^Comparison between the combined group and the LAAC-only group. Values are mean ± SD or *n* (%) as appropriate. AF, atrial fibrillation; BMI, body mass index; CA, catheter ablation; LAA, left atrial appendage; LAAC, left atrial appendage closure; LVEF, left ventricular ejection fraction; TIA, transient ischemic attacks; TE, thromboembolism.

**Table 2:** Baseline characteristics of the study population after propensity score matching.

	Combined (*n* = 76)	CA-only (*n* = 76)	*P* ^*∗*^	LAAC-only (*n* = 76)	*P* ^†^
Female	37 (48.7)	40 (52.6)	0.626	38 (50.0)	0.871
Age (years)	69.9 ± 7.9	69.5 ± 7.8	0.703	71.36 ± 8.9	0.300
BMI (kg/m^2^)	24.6 ± 3.2	24.9 ± 3.7	0.543	24.3 ± 3.3	0.644
Paroxysmal AF	37 (48.7)	38 (50.0)	0.871	27 (35.5)	0.100
Persistent AF	39 (51.3)	38 (50.0)	0.871	49 (64.5)	0.100
Coronary artery disease	16 (21.1)	15 (19.7)	0.840	18 (23.7)	0.426
Hypertension	56 (73.7)	54 (71.1)	0.717	57 (75.0)	0.853
Heart failure	23 (30.3)	21 (27.6)	0.721	19 (25.0)	0.468
Diabetes mellitus	14 (18.4)	15 (19.7)	0.836	15 (19.7)	0.836
Previous stroke/TIA/TE	23 (30.3)	15 (19.7)	0.134	20 (26.3)	0.589
Left atrial diameter (mm)	42.7 ± 5.7	41.7 ± 4.9	0.226	44.1 ± 5.9	0.163
LVEF (%)	63.9 ± 6.3	64.2 ± 5.4	0.746	62.7 ± 6.9	0.273
CHA_2_DS_2_-VASc score	3.6 ± 1.3	3.4 ± 1.4	0.484	3.7 ± 1.4	0.719
HAS-BLED score	3.3 ± 1.1	2.6 ± 0.9	<0.001	3.4 ± 1.1	0.413

*∗*Comparison between the combined group and the CA-only group. ^**†**^Comparison between the combined group and the LAAC-only group. Values are mean ± SD or *n* (%) as appropriate. AF, atrial fibrillation; BMI, body mass index; CA, catheter ablation; LAA, left atrial appendage; LAAC, left atrial appendage closure; LVEF, left ventricular ejection fraction; TIA, transient ischemic attacks; TE, thromboembolism.

**Table 3:** Catheter ablation and arrhythmia follow-up.

	Combined (*n* = 76)	CA-only (*n* = 76)	*P*
Mapping system			
CARTO	33 (43.4)	37 (48.7)	0.515
EnSite	43 (56.6)	39 (51.3)	0.515
PVI only	39 (51.3)	41 (53.9)	0.745
PVI plus linear/CFAE ablation	37 (48.7)	35 (46.1)	0.745
Cardioversion	36 (47.4)	32 (42.1)	0.514
Procedure time^*∗*^ (min)	129 ± 24.5	133.1 ± 26.4	0.388
Fluoroscopy time^*∗*^ (min)	5.4 ± 2.4	5.9 ± 2.6	0.336
Complications	3 (3.9)	2 (2.6)	0.650
Pericardial effusion	1 (1.3)	1 (1.3)	1.000
Stroke	0 (0.0)	0 (0.0)	—
Major bleeding events	0 (0.0)	0 (0.0)	—
Death	0 (0.0)	0 (0.0)	—
Complications of vascular access	2 (2.6)	1 (1.3)	0.561
Average follow-up (months)	24.0 ± 5.2	23.7 ± 4.9	0.717
AF-free follow-up			
Overall	51 (67.1)	53 (69.7)	0.727
Paroxysmal AF	26 (70.3)	29 (76.3)	0.554
Persistent AF	25 (64.1)	24 (63.2)	0.931
Redo ablation	12 (15.8)	9 (11.8)	0.481
Follow-up events	3 (3.9)	4 (5.3)	0.699
Ischemic stroke events	1 (1.3)	1 (1.3)	1.000
Bleeding events	2 (2.6)	3 (3.9)	0.650

^*∗*^The procedure and fluoroscopy time were calculated from femoral venous puncture to the end of the ablation. Values are mean ± SD or *n* (%) as appropriate. AF, atrial fibrillation; CA, catheter ablation; CFAE, complex fragmented atrial electrogram; PVI, pulmonary vein isolation.

**Table 4:** Left atrial appendage closure and device follow-up.

	Combined (*n* = 76)	LAAC-only (*n* = 76)	*P*
Morphology of LAA			
Cauliflower	48 (63.2)	52 (68.4)	0.494
Chicken wing	16 (21.1)	8 (10.5)	0.075
Cactus	6 (7.9)	9 (11.8)	0.415
Windsock	6 (7.9)	7 (9.2)	0.772
Procedure time^*∗*^ (min)	26.1 ± 6.4	28.0 ± 8.7	0.149
Fluoroscopy time^*∗*^ (min)	4.9 ± 1.9	4.5 ± 2.4	0.273
LAA ostium width (mm)	22.9 ± 3.1	23.5 ± 3.4	0.220
Device size (mm)	29.0 ± 3.0	29.1 ± 3.2	0.876
Number of device size changes per patient			
0	73 (96.1)	71 (93.4)	0.468
1	3 (3.9)	5 (6.6)	0.468
2	0 (0.0)	0 (0.0)	—
Device compression (%)	19.6 ± 4.5	18.4 ± 4.3	0.085
Successful implantation	76 (100)	76 (100)	—
Peridevice leak at implantation			
Complete occlusion of LAA	72 (94.7)	70 (92.1)	0.513
Leak ≤5 mm	4 (5.3)	6 (7.9)	0.513
Leak >5 mm	0 (0.0)	0 (0.0)	—
Complications	3 (3.9)	2 (2.6)	0.650
Pericardial effusion	1 (1.3)	0 (0.0)	0.317
Stroke	0 (0.0)	0 (0.0)	—
Coronary air embolism	0 (0.0)	1 (1.3)	0.317
Bleeding	0 (0.0)	0 (0.0)	—
Death	0 (0.0)	0 (0.0)	—
Complications of vascular access	2 (2.6)	1 (1.3)	0.561
Peridevice leak at 45 days			
Complete occlusion of LAA	63 (82.9)	65 (85.5)	0.656
New peridevice leaks	10 (13.2)	7 (9.2)	0.440
Persistent peridevice leaks	3 (3.9)	4 (5.3)	0.699
Device-associated thrombosis	0 (0.0)	1 (1.3)	0.317
Average follow-up (months)	24.0 ± 5.2	24.1 ± 4.9	0.961
Follow-up events	3 (3.9)	2 (2.6)	0.650
Ischemic stroke events	1 (1.3)	0 (0.0)	0.317
Bleeding events	2 (2.6)	2 (2.6)	1.000

^*∗*^The procedure and fluoroscopy time were calculated from the WATCHMAN access sheath exchange to the end of the procedure. Values are mean ± SD or *n* (%) as appropriate. AF, atrial fibrillation; LAA, left atrial appendage; LAAC, left atrial appendage closure.

## Data Availability

The data used to support the findings of this study are available from the corresponding author upon request.
